# Towards a fair consideration of PrEP as part of combination HIV prevention in Latin America

**DOI:** 10.7448/IAS.19.7.21113

**Published:** 2016-10-18

**Authors:** Giovanni Ravasi, Beatriz Grinsztejn, Ricardo Baruch, Juan Vicente Guanira, Ricardo Luque, Carlos F Cáceres, Massimo Ghidinelli

**Affiliations:** 1 Pan American Health Organization (PAHO), Washington, DC, USA; 2 Instituto Nacional de Infectologia Evandro Chagas, Fundação Oswaldo Cruz, Rio de Janeiro, Brazil; 3 Instituto Nacional de Salud Pública, Cuernavaca, México; 4 Investigaciones Medicas en Salud, Lima, Peru; 5 Ministerio de Salud y Protección Social, Bogotá, Colombia; 6 Centro de Investigación Interdisciplinaria en Sexualidad, SIDA y Sociedad, Universidad Peruana Cayetano Heredia, Lima, Peru

**Keywords:** pre-exposure prophylaxis, HIV, prevention, antiretrovirals, Latin America

## Abstract

**Introduction:**

Despite progress in scaling up antiretroviral treatment, HIV prevention strategies have not been successful in significantly curbing HIV incidence in Latin America. HIV prevention interventions need to be expanded to target the most affected key populations with a combination approach, including new high impact technologies. Oral pre-exposure prophylaxis (PrEP) is recommended as additional prevention choice for individuals at higher risk of infection and could become a cost-effective prevention tool. We discuss the barriers and solutions for a fair consideration of PrEP as part of combination HIV prevention strategies in Latin America.

**Discussion:**

Although demonstration projects are ongoing or being planned in a number of countries, to date no Latin American country has implemented a public PrEP programme. The knowledge of policymakers about PrEP implementation needs to be strengthened, and programmatic guidance and cost estimate tools need to be developed to support adequate planning. Despite high levels of awareness among health providers, especially if engaged in HIV or key population care, willingness to prescribe PrEP is still low due to the lack of national policies and guidelines. Key populations, especially men who have sex with men, transgender women and sex workers, have been engaged in demonstration projects, and qualitative research shows high awareness and willingness to use PrEP, especially if accessible in the public sector for free or at affordable price. Concerns of safety, adherence, effectiveness and risk compensation need to be addressed through targeted social communication strategies to improve PrEP knowledge and stimulate demand. Alliance among policymakers, civil society and representatives from key populations, healthcare providers and researchers will be critical for the design and successful implementation of PrEP demonstration projects of locally adapted delivery models. The use of mechanisms of joint negotiation and procurement of antiretrovirals could reduce costs and significantly increase the cost-effectiveness of PrEP.

**Conclusions:**

PrEP is an additional prevention tool and should be implemented in combination and synergy with other prevention interventions. PrEP programmes should target high-risk individuals from key populations for higher cost-effectiveness. Demonstration projects may generate strategic information for and lead to the implementation of full-scale PrEP programmes.

## Introduction

By the end of 2014, Latin America had achieved substantial results in expanding antiretroviral treatment (ART) coverage to 47% of estimated people living with HIV [1]. However, progress in *halting and beginning to reverse the HIV epidemic by 2015* [2] has been less successful, and despite some progress (13% reduction between 2000 and 2014 in the estimated number of new HIV cases per year), the region fell short against expected targets [1,3]. Considering current trends in HIV incidence, Latin American countries will need to accelerate the scale-up of ART to fully benefit from the preventive effect of treatment, as well as expand HIV combination prevention interventions, targeting key populations and including new effective technologies [4].

Among newly available tools, daily oral pre-exposure prophylaxis (PrEP) with tenofovir/emtricitabine (TDF/FTC) has been proven safe and effective to prevent HIV among men who have sex with men (MSM), transgender women (TGW), heterosexuals at high risk, uninfected partners in serodiscordant couples and injection drug users [5–11]. In 2014, the World Health Organization (WHO) released a recommendation on the use of oral PrEP as additional HIV prevention choice for MSM within a comprehensive HIV prevention approach, reviewed in 2015 to expand PrEP indication to all people at substantial risk of HIV infection [12,13].

To date, PrEP demonstration projects have been expanded worldwide; however the United States remains the only country in the Americas to have started large-scale implementation of PrEP regulated by normative bodies and based on clinical guidelines [14–16].

Considering that the HIV epidemic in Latin American countries is mainly concentrated in key populations, frequently hidden epidemics involving hard-to-reach individuals with inequitable access to services and victims of stigma, discrimination and criminalization in some cases, HIV prevention interventions should be focused, acceptable and tailored to the needs of these most affected groups [17,18]. As part of HIV combination prevention packages, PrEP could become a cost-effective tool, especially when used by individuals at higher risk of infection and in addition to traditional prevention interventions. Nevertheless, implementing PrEP in resource-limited settings entails several challenges related to demand, service delivery, regulatory aspects and affordability of medications [19].

The purpose of this paper is to discuss the barriers and solutions for a fair consideration of PrEP as part of combination HIV prevention policies and programmes in Latin America.

## Discussion

### Current status of PrEP in Latin America

Since the implementation of the first proof-of-concept multicountry trial in MSM, which included sites in Peru, Brazil and Ecuador, an open-label follow-up continued enrolling MSM and TGW to evaluate PrEP uptake, adherence, safety and effectiveness [20,21]. Despite the contribution of Latin American countries to these trials, policy development and programming have shown little progress, and no country has yet launched public PrEP services.

Brazil is currently carrying out multiple demonstration projects. The PrEP Brasil Project (clinical trial NCT01989611), coordinated by the Oswaldo Cruz Foundation, initially implemented in Rio de Janeiro and São Paulo in partnership with non-governmental organizations (NGOs), is expanding to additional sites in Porto Alegre and Manaus (Grinsztejn, private communication). The objective of this project is to assess the uptake, safety and feasibility of free access to PrEP for high-risk MSM and TGW, aiming at generating information for the subsequent PrEP implementation in the public sector. In addition, a demonstration project in MSM, sex workers and drug users is being planned to start in 2016 in multiple sites (São Paulo, Porto Alegre, Ribeirão Preto and Fortaleza) [22].

In Peru, oral PrEP is prescribed on demand to a few MSM who can purchase antiretroviral (ARV) medications (Guanira, personal communication), and a larger demonstration project among MSM and TGW is being planned at three sites (i.e. Lima-Callao, Northern Coast and Amazon) in late 2016 (Caceres, personal communication). Other PrEP projects are being implemented in Mexico City at Clínica Condesa, where PrEP is offered to a limited number of male sex workers and other MSM (Baruch, private communication), and Guatemala City by the NGO Amigos Contra el SIDA (Ghidinelli, private communication).

The cautious attitude of Latin American countries *vis-à-vis* the implementation of PrEP was also reflected in the 2015 Rio de Janeiro Call to Action, a consensus statement endorsed by representatives from governments, academia, health services and civil society from 26 Latin American and Caribbean countries. This statement includes regional goals and targets for HIV prevention, one of which refers to the implementation of PrEP projects in 10 countries by 2020 [23].

The introduction of PrEP requires engaging policymakers. Qualitative research conducted in Peru in 2010 to 2011 showed that less than 50% of policymakers interviewed were aware of PrEP [24]. Although recognizing its value as a prevention tool for people at higher risk, policymakers highlighted concerns about risk compensation, adherence, effectiveness and side effects.

Awareness and willingness to provide PrEP services among healthcare providers is critical. In a survey conducted in Peru in 2012, 57.5% of providers were aware of PrEP, although awareness was higher in ART prescribers (81.3%), in those that were providing care to more than 50 MSM (80.4%), or to at least 50 HIV-positive persons (77.1%). On the other hand, only 44.6% reported willingness to prescribe PrEP, and the likelihood increased if national policies and guidelines were issued (70.3%), if more evidence on effectiveness (68.5%) and more friendly regimens were available (62.2%) [25]. Health providers from Peru and Brazil involved in the early stages of PrEP trials reported concerns about adherence and risk compensations, as well as doubts about the support from national health authorities [26]. Another barrier on the health provider side may be the discriminatory attitude towards members of key populations, in particular MSM and TGW, which may prevent individuals from accessing services or openly discussing their needs and therefore receiving adequate care [27].

Finally, awareness, acceptability and willingness to use PrEP among target populations is key to shape the demand for PrEP. Research conducted in 2015 among 68 MSM in urban sites in Mexico showed that 100% of participants were aware of PrEP and 70% were willing to use it if available for free or offered at an affordable price (Baruch et al. Unpublished data). In Brazil, although approximately 60% of 734 MSM from Rio de Janeiro interviewed in 2014 to 2015 were aware of PrEP, 95% stated they would be very interested in using it if it were available in the public sector [28]. In Peru, a survey conducted in 2011 among MSM, TGW and female sex worker (FSW) showed very limited awareness about PrEP, with the exception of few individuals who had taken part in the initial iPrEX study [29], but subsequent surveys showed that 91% MSM and FSW were willing to use PrEP and adopt it as soon as it became available [30] and that MSM and TGW had high acceptance of either oral (96.2%) or rectal (91.7%) PrEP administration [31]. Across studies, common concerns about PrEP from key populations were related to safety, dosing, route of administration, adherence and effectiveness, as well as costs and acceptable service delivery [32]. Peruvian MSM and TGW showed concerns about risk compensation, although Brazilian MSM and Peruvian FSW understood that PrEP does not substitute condoms nor prevent other sexually transmitted infections (STIs) [26,29–31].

Additional challenges for the implementation of PrEP programmes in Latin America are related to the cost of medicines and regulatory aspects. Many Latin American countries have been utilizing mechanisms of joint price negotiation and procurement of ARVs for ART programmes with significant price reduction; for example, the generic fixed-dose combination of TDF/FTC is currently available through the Strategic Fund (SF) of the Pan-American Health Organization (PAHO) at a price of US$5.37 per pack (30 tabs) [33]. However, not all countries in the region are signatories of the SF or are using it to purchase ARVs, and prices continue to be higher in countries like Mexico and Chile [34]. FDA approval of the TDF/FTC combination for preventive use was granted in 2012 [35]. Since then Gilead Science has applied for approval in countries worldwide, including Brazil. Nevertheless, approval by the Brazilian Drug Regulatory Authority is still on hold and to date in Latin America only Peru has registered TDF/FTC for prevention use [36,37].

### The potential role of PrEP for HIV prevention in Latin America

Gaps still exist in the extent and consistency of condom use in key populations in Latin America: 65% of FSW report using a condom with the last client (median based on data from 21 countries, 2011 to 2014); 47% of MSM report using a condom in the last episode of anal sex (median based on data from 30 countries, 2011 to 2014) [38].

Considering that MSM frequently engage in unprotected anal intercourse [39–42], MSM who engage in risky sexual behaviour, but also male sex workers and TGW, could be candidates for the initial phase of implementation of PrEP programmes. The observation that higher willingness to use PrEP among MSM was significantly associated with exclusive or more frequent receptive anal sexual behaviour [31], history of unprotected anal intercourse with more than two men [28] and perceived risk of HIV infection [32] also supports this approach. In addition, MSM, TGW and FSW recognized the potential value of PrEP as protection in case of condom rupture or non-availability, or in case of unprotected casual sex [29].

Mathematical models provide further evidence in support of PrEP programmes targeting key populations. A study conducted in Peru to assess the population-level impact, cost and cost-effectiveness of PrEP in MSM and TGW in Lima showed that with a relatively low PrEP coverage (5%), over 8% of new infections could be averted if individuals at higher risk were prioritized (TGW and sex workers) and guaranteeing adherence levels observed in clinical trials. However, when the averted downstream costs of ARVs are included in the model, most PrEP scenarios become cost-effective [43]. If we compare the price of TDF/FTC used in mathematical modelling (US$420 to 600 per year for Truvada [41]) with the regional reference price of the generic TDF/FTC combination (approximately US$65 per year through PAHO SF [33]; plus 15% for shipment and insurance), the potential cost-effectiveness of PrEP in high-risk individuals could be enormously improved with the use of generics.

### Strategies to overcome barriers and support PrEP programme implementation

PrEP awareness among policymakers, in particular national HIV programme managers, has certainly increased over time [23]. Nevertheless, to advance programme implementation, their knowledge of PrEP still needs to be strengthened and practical toolkits and guidance documents to support the operationalization of PrEP programmes and the design of demonstration projects to phase in PrEP need to be developed and disseminated.

Implementing PrEP may require in most countries an initial phase of formative assessment to define target populations and criteria to identify PrEP candidates at higher risk, assess acceptability and likely uptake of PrEP, as well as a demonstration project to test feasibility and effectiveness of locally adapted models of delivery. For the design of PrEP delivery models, the alliance and collaboration among policymakers, civil society and representatives from key populations, health service providers and research groups will be critical.

The experience gained from demonstration projects will provide strategic information for the full-scale implementation of PrEP. Modelling tools for the projection of population-level estimates of PrEP needs, as well as other programmatic costing tools, will contribute to more accurate budgeting and planning.

In addition, strengthening the PrEP literacy of potential users through targeted social communication strategies will be critical to disseminate information about the purpose and rationale for the use of ARV medicines to prevent HIV (not STIs), eligibility criteria, dosing and monitoring requirements, and the combination with other preventive tools for greater preventive benefit. Such strategies will stimulate demand for PrEP among most appropriate candidates (i.e. uninfected individuals from key populations at higher risk for HIV acquisition), minimize expectations and demand from people not at higher risk, and ensure the successful introduction of cost-effective PrEP programmes.

Furthermore, national protocols on the use of PrEP need to be developed for healthcare providers, together with comprehensive training packages that include aspects of HIV risk-assessment for PrEP eligibility, as well as prescription, delivery and monitoring guidelines. Provision of person-centred care for key populations, taking into account their needs and vulnerabilities, addressing aspects of sexual orientation and gender identity, sexual behaviour and sexual health in a stigma- and discrimination-free environment, should also be included in training packages to promote access to care in key populations and improve the assessment of individuals that will most likely benefit from PrEP and the overall cost-effectiveness of PrEP programmes [44].

The implementation of PrEP programmes should be integrated within existing HIV prevention services for key populations to minimize the need for additional resources while ensuring access to the comprehensive package of services that safe and effective PrEP implementation requires: periodic HIV screening, renal function monitoring, adherence counselling, promotion of STI/HIV prevention and provision of condoms and lubricants, and effective and timely linkage to care in case HIV infection occurs [6,45].

Finally, the feasibility and sustainability of PrEP programmes also requires minimizing the financial burden of the procurement of ARVs (TDF/FTC), especially as national programmes face the challenge of decreased donor funding and increased domestic expenditure for ARV medicines while adopting the new WHO “treat all” recommendation. The anticipated financial burden of PrEP programmes may be another factor for the cautious approach to PrEP by policymakers. In contrast, joint negotiations and pooled procurement mechanisms, such as the PAHO SF, may offer an opportunity for more efficient use of resources and significant savings by acquiring WHO prequalified generic ARVs for both ART and PrEP at competitive prices.

### PrEP implementation research needs

Through the implementation of demonstration projects, as well as monitoring and evaluation of the several local initiatives of PrEP delivery currently underway, it will be possible to generate more evidence on the most effective service-delivery models in the context of different types of health services (e.g. primary level, specialized facilities, STI clinics, etc.), community-based services or a combination of both, as well as models based on public-private partnership. Implementation research should also assess the effectiveness of PrEP prescription and monitoring by medical providers (e.g. specialist physician vs. generalists), compared to models based on task-shifting to other health cadres or lay providers, especially in case of community NGO-based models.

In addition, demonstration projects will offer an opportunity to address additional research topics, such as integration of STI screening and management, alternative testing options (e.g. mobile testing; self-testing) and non-daily regimes (e.g. “on demand” PrEP), as well as knowledge gaps (e.g. long-term effectiveness and safety, topical and injected administration, use of other ARVs for prevention, etc.). Participating in global and regional research networks will assure that research methodologies are harmonized for comparison among countries and compilation of data for regional and global analysis.

## Conclusions

Considering the epidemiological context of the HIV epidemic in Latin America and the available evidence from cost-effectiveness studies, we anticipate that PrEP implementation may achieve higher impact on HIV prevention when targeting individuals at significantly higher risk of HIV exposure within most affected key populations identified in each country.

If Latin American countries intend to pursue the goal of ending AIDS as a public health problem by 2030, HIV combination prevention strategies need to be expanded and focused on the most affected populations, tailored to their needs and updated to include all available innovative tools of proven efficacy. PrEP is certainly one additional option and should be implemented in combination and synergy with condom/lubricant programmes and traditional behaviour change strategies, while addressing the unfinished business of scaling up HIV testing and access to ART. Finally, addressing criminalization, stigma and discrimination against key populations is an overarching imperative to create an enabling environment to ensure equitable access to the whole range of HIV prevention, care and treatment services.
